# Epidermal Growth Factor Receptor Cell Survival Signaling Requires Phosphatidylcholine Biosynthesis

**DOI:** 10.1534/g3.116.034850

**Published:** 2016-09-07

**Authors:** Matt Crook, Awani Upadhyay, Liyana J. Ido, Wendy Hanna-Rose

**Affiliations:** Department of Biochemistry and Molecular Biology, The Pennsylvania State University, University Park, Pennsylvania 16802

**Keywords:** TRPV, necrosis, phospholipid, *pmt-1*, *pnc-1*

## Abstract

Identification of pro-cell survival signaling pathways has implications for cancer, cardiovascular, and neurodegenerative disease. We show that the *Caenorhabditis elegans* epidermal growth factor receptor LET-23 (LET-23 EGFR) has a prosurvival function in counteracting excitotoxicity, and we identify novel molecular players required for this prosurvival signaling. uv1 sensory cells in the *C. elegans* uterus undergo excitotoxic death in response to activation of the OSM-9/OCR-4 TRPV channel by the endogenous agonist nicotinamide. Activation of LET-23 EGFR can effectively prevent this excitotoxic death. We investigate the roles of signaling pathways known to act downstream of LET-23 EGFR in *C. elegans* and find that the LET-60 Ras/MAPK pathway, but not the IP_3_ receptor pathway, is required for efficient LET-23 EGFR activity in its prosurvival function. However, activation of LET-60 Ras/MAPK pathway does not appear to be sufficient to fully mimic LET-23 EGFR activity. We screen for genes that are required for EGFR prosurvival function and uncover a role for phosphatidylcholine biosynthetic enzymes in EGFR prosurvival function. Finally, we show that exogenous application of phosphatidylcholine is sufficient to prevent some deaths in this excitotoxicity model. Our work implicates regulation of lipid synthesis downstream of EGFR in cell survival and death decisions.

Epidermal growth factor receptor (EGFR) signaling is well studied in terms of its growth and differentiation functions. However, EGFR plays other roles in cell physiology that are not as well elucidated. In particular, EGFR regulates cell responses governing survival and death. Survival signaling by EGFR has been observed (Henson and Gibson 2006), and prosurvival signaling mechanisms revealed in cell culture studies involve both suppression of proapoptotic protein expression and activation of antiapoptotic protein expression (Bergmann *et al.* 2002; Gibson *et al.* 2002; Henson *et al.* 2003). Genetic knockout studies in mice demonstrate that loss of EGFR activity results in neural degenerative phenotypes that suggest survival functions for EGFR signaling in the brain (Kornblum *et al.* 1998; Sibilia *et al.* 1998).

In contrast to the survival signaling functions, genetic experiments in *Caenorhabditis elegans* clearly demonstrate proapoptotic function of EGFR signaling (Jiang and Wu 2014), and EGF has also been shown to cause apoptosis in some cell types in culture (Armstrong *et al.* 1994; Gulli *et al.* 1996; Garcia *et al.* 2006). Given the variety of known signaling pathways that can be activated by EGFR, it is not surprising that the role of EGFR signaling in cell death is complex and thus not fully defined.

The *C. elegans* system has proven valuable for both revealing EGFR functions and deciphering signaling mechanisms. LET-23 is the sole *C. elegans* EGFR. Numerous roles have been ascribed to LET-23, and the signaling pathways that mediate LET-23 EGFR activity in each function have been at least partially delineated. LET-23 EGFR is required for fate specification of vulval epithelial cells and four mechanosensory cells, called uv1 cells, in the ventral uterus (Chang *et al.* 1999; Moghal and Sternberg 2003). These EGFR-mediated differentiation signals, as well as the signals that drive apoptosis in response to LET-23 activation in *C. elegans*, require the conserved LET-60 Ras/MAPK pathway and a transcriptional output involving the transcriptional effector LIN-1 (Chang *et al.* 1999; Moghal and Sternberg 2003; Jiang and Wu 2014). LET-60 also mediates the effect of LET-23 EGFR in morphogenesis of the excretory system (Parry and Sundaram 2014). In other biological contexts, LET-23 EGFR signals via alternative pathways. LET-23 EGFR promotes ovulation and maintenance of health span by activating the phospholipase encoded by PLC-3 and the IP_3_ receptor ITR-1 (Clandinin *et al.* 1998; Yin *et al.* 2004; Iwasa *et al.* 2010; Yu and Driscoll 2011). And finally, PLC-3 and diacylglycerol binding proteins mediate LET-23 EGFR function in behavioral quiescence (Van Buskirk and Sternberg 2007). We have revealed a protective role for LET-23 signaling in antagonizing excitotoxic death in *C. elegans* and uncovered a novel mechanism required for signaling.

Excitotoxic cell death is a pathological response to conditions that hyperactivate ion channels in the nervous system, resulting in an ion imbalance and a necrotic-like death (Sattler and Tymianski 2001; Fujikawa 2015). The uv1 cells in the *C. elegans* uterus stimulate egg laying in response to stretching of the uterus upon accumulation of eggs (Jose *et al.* 2007). High levels of the nicotinamide (NAM) form of vitamin B_3_, produced by disrupting the function of the nicotinamidase PNC-1 or exogenous application of NAM, hyperactivate a TRPV cation channel and cause excitotoxic death of the uv1 cells (Huang and Hanna-Rose 2006; Vrablik *et al.* 2009; Upadhyay *et al.* 2016). Surprisingly, this excitotoxic death is efficiently suppressed by gain-of-function mutations in *let-23*, as well as by overexpression of the LET-23 ligand LIN-3 EGF (Huang and Hanna-Rose 2006). We have explored the signaling mechanisms used by LET-23 in this prosurvival role by exploring the roles of known downstream mediators of LET-23 signaling in *C. elegans* and by screening for additional signaling molecules. We demonstrate that while signaling through LET-60 Ras is required for LET-23 survival signaling, activation of LET-60 Ras or MAPK is not sufficient to mediate survival and that PLC-3 signaling is dispensable for LET-23 survival signaling. Finally, we show that phosphatidylcholine biosynthesis is necessary and partially sufficient to mediate LET-23 survival function.

## Materials and Methods

### C. elegans strains and maintenance

Strains were maintained under standard conditions at 20° (Brenner 1974) using *Escherichia coli* OP50 as a food source. In supplementation experiments, solutions were added to preseeded culture plates and allowed to be absorbed into the agar overnight. Animals were supplemented with 25 mM NAM (Alfa Aesar, Ward Hill, MA), 30 mM choline (Fluka, St. Louis, MO), or 30 mM cytidine diphosphate choline (CDP-choline; Alfa Aesar) by diluting from filter-sterilized 1 M stock solutions prepared in water. Animals were also supplemented with 30 µM L-α-phosphatidylcholine (PtdCholine) (Sigma Aldrich, St. Louis, MO) by diluting from a filter-sterilized 10 mM working solution prepared in water, which was in turn diluted from a 0.5 M stock solution prepared in 100% ethanol. An equal volume of 2% ethanol was applied to plates used as controls in the PtdCholine supplementation experiments. Strains used in this work are listed in Table 1.

**Table 1:** Strains used in this study

Strain	Genotype
BL5715	II *inIs179 (ida-1*::*gfp)*
HV285	II *inIs179 (ida-1*::*gfp)*; IV *let-60(n1046*gf*)*
HV330	II *let-23(sa62*gf*) unc-4(e120)*; IV *pnc-1(ku212)*; Ex(*ida-1*::*GFP*)
HV560	II *inIs179 (ida-1*::*gfp)*; IV *pnc-1(pk9605)*
HV607	lI *let-23(sa62*gf*) unc-4(e120)*; Ex[ida-1::gfp]
HV623	II *inIs179 (ida-1*::*gfp)*; IV *let-60(n1046*gf*) itr-1(sy290*gf*) unc-24(e138)*
HV639	II *inIs179 (ida-1*::*gfp)*; IV *pnc-1(pk9605) let-60(n1046*gf*) itr-1(sy290*gf*) unc-24(e138)*
HV651	II *inIs179 (ida-1*::*gfp)*; IV *itr-1(sy290*gf*) unc-24(e138)*
HV662	II *inIs179 (ida-1*::*gfp)*; IV *pnc-1(pk9605) let-60(n1046*gf*)*
HV663	II *inIs179 (ida-1*::*gfp)*; IV *pnc-1(pk9605) itr-1(sy290*gf*) unc-24(e138)*
HV760	II *let-23(sa62*gf*) inIs179 (ida-1*::*gfp)*
HV776	II *let-23(sa62*gf*) unc-4(e120) inIs179 (ida-1*::*gfp)*; IV *pnc-1(pk9605)*
PJ1063	IV *let-60(ga89*gf); V *ccIs55*
PJ1115	IV *gaIs37*[EF1α-D-mek(gf); hs-mpk-1(gf)]; V ccIs55

### Phenotypic analysis: uv1 survival and specification

uv1 cell survival was scored and calculated using two methods. In some experiments, healthy GFP-positive uv1 cells were counted using an integrated *ida-1*::*gfp* transgene, which results in GFP expression in all four uv1 cells in wild-type animals (Zahn *et al.* 2001). Percentage uv1 survival or specification was calculated using the following formula: number of GFP-positive cells observed/number of cells expected × 100. In other experiments where the integrated GFP marker was not available in the strain, uv1 cell corpses were counted, and the percentage uv1 survival was calculated using the following formula: (number of cells expected − number of corpses observed)/number of cells expected. Number of cells expected is calculated by multiplying the number of animals examined by 4 (the typical number of uv1 cells per animal) or by the appropriate multiplier, as determined in Table 2.

**Table 2:** LET-23 EGFR and LET-60 Ras induce ectopic uv1 specification

Genotype	No. of GFP-Positive uv1 Cells				Average No. of uv1 Cells Specified	*n*
	7	6	5	4		
Wild-type (strain BL5715)	0	0	0	60	4	60
*let-23(sa62*gf*)* (strain HV760)	0	1	8	49	4.17*	58
*let-60(n1046*gf*)* (strain HV285)	1	29	55	51	4.85***	136
*itr-1(sy290*gf*)* (strain HV651)	0	0	0	111	4	111
*let-60(n1046*gf*) itr-1(sy290*gf*)* (strain HV623)	1	38	71	19	5.16**^,^***	129

*n*, number of animals examined.

* *P* < 0.005.

** *P* < 0.001, Student’s *t*-test, comparison to *let-60*(*n1046*gf).

*** *P* < 0.001, Student’s *t*-test, comparisons to wild type.

### RNAi

RNAi clones, with the exception of *pmt-2*, were from the *C. elegans* RNAi Library (Source BioScience, Nottingham, UK). Constructs were confirmed by sequencing. *pmt-2* RNAi was constructed by amplifying an 854 bp fragment, using primers described previously (Palavalli *et al.* 2006), ligating the fragment into the L4440 T7 vector (Addgene plasmid no. 1654) and transforming into RNAi feeding strain HT115. RNAi feeding assays were carried out as described (Ahringer 2006). Briefly, RNAi bacterial cultures were spotted onto Nematode Growth Medium (NGM) 25 μg/ml^−1^ carbenicillin 1 mM Isopropyl β-D-1-thiogalactopyranoside (IPTG) plates and allowed to grow at room temperature for 3 to 5 d. Five to ten L4s were placed onto each plate and incubated at 20° until their L4 offspring could be scored.

### Data and reagent availability

The authors declare that the data supporting the findings of this study are available within the article and its Supplemental Material, Table S1. Strains and reagents are available upon request.

## Results

### Ras/MAPK signaling is necessary but not sufficient for let-23(sa62gf) suppression of excitotoxic cell death

**Figure 1:**
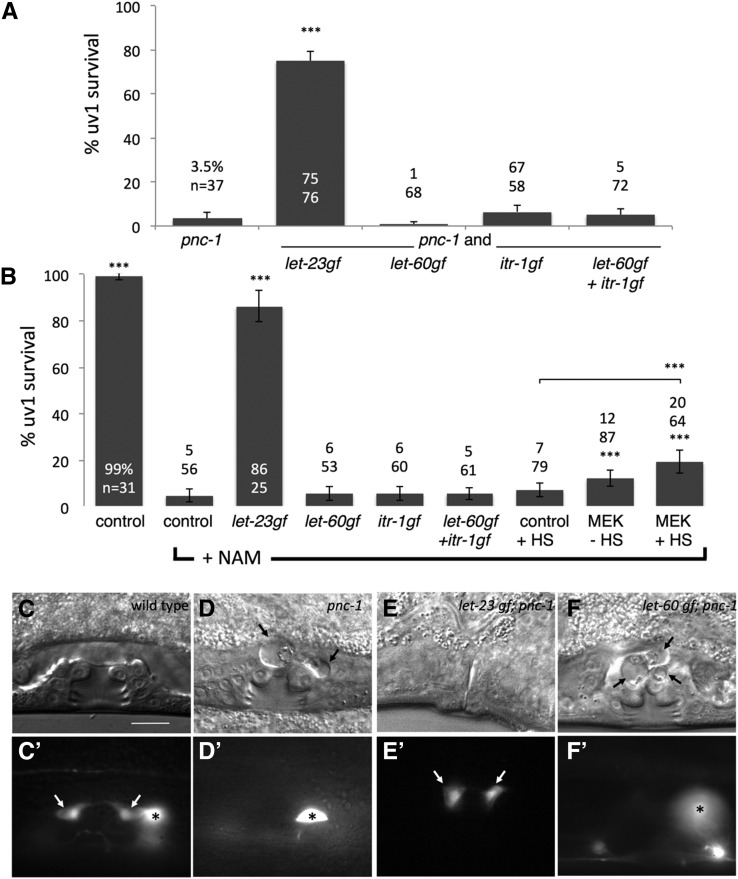
Activation of known signaling pathways downstream of LET-23 fails to mimic activation of LET-23 in cell survival signaling. (A) While *let-23*(*sa62*gf) promotes uv1 cell survival in *pnc-1* mutants, neither the *let-60*(*n1046*) nor *itr-1*(*sy290*) gain-of-function alleles, alone or in combination, are sufficient to provide a cell survival signal in *pnc-1* mutants. Strains used are HV560, HV776, HV662, HV663, and HV639. (B) Similarly, *let-23*(*sa62*gf) promotes uv1 cell survival in animals supplemented with 25 mM nicotinamide (NAM). However, neither the *let-60*(*n1046*) nor *itr-1*(*sy290*) gain-of-function alleles, alone or in combination, nor activation of MAP kinases, is sufficient to provide a full cell survival signal in NAM-supplemented animals. Strains used are BL5715 (control), HV607, HV285, HV651, HV623, and PJ1115. Percentage uv1 cells surviving and number of animals examined are indicated on or above each bar in panel (A and B). Error bars are 95% C.I. Asterisks indicate statistical tests performed via comparison to *pnc-1* in (A) and relative to control plus NAM or relative to control + heat shock (HS) in (B). *** *P* < 0.001, calculated using Fisher’s exact test with Bonferroni correction considering four pairwise comparisons for (A) and 10 pairwise comparisons for (B). (C–F) DIC and corresponding fluorescence photomicrographs for animals carrying the *inIs179*[*ida-1*::*GFP*] transgene, a uv1 cell marker. uv1 cells are marked with arrows and the unrelated GFP-positive HSN cell is marked with an asterisk in all fluorescence panels. Scale bar is 10 μm. (C, C′) Wild-type (strain BL5715) L4 animal with a normal mid-L4 stage vulva and no uv1 cell corpses (C) and two GFP-positive uv1 cells (C′). (D, D′) *pnc-1* (strain HV560) L4 animal with a mid-L4 stage vulva with two visible uv1 cell corpses (arrows in D) and no GFP-positive uv1 cells (D′). (E, E′) *let-23*(*sa62*gf) *pnc-1* (strain HV776) young adult animal. Normal vulval eversion has already occurred. The animal has no uv1 cell corpses (E) and two GFP-positive uv1 cells (E′). We documented an older animal in these panels to demonstrate the lack of death even at later stages. (F, F′) *let-60* (*n1046*gf) *pnc-1* (strain HV662) L4 animal with a mid-L4 stage vulva with three visible uv1 cell corpses (arrows in F) and no GFP-positive uv1 cells (F′). The small GFP-positive cells on the ventral surface of the animal are *ida-1*::*GFP*-positive ventral cord neurons.

NAM is an agonist for the OSM-9/OCR-4 TRPV channel (Upadhyay *et al.* 2016). We have previously shown that accumulation of NAM caused by mutation of the nicotinamidase PNC-1 or direct supplementation of *C. elegans* cultures induces excitotoxic death in the cells that express the OSM-9/OCR-4 channel, including the uv1 cells (*e.g.*, Figure 1) (Upadhyay *et al.* 2016). Notably, activation of LET-23 EGFR via the gain-of-function allele *let-23*(*sa62*gf) effectively prevents death of the uv1 cells caused by mutation of *pnc-1* or by supplementation of wild-type cultures with NAM (Huang and Hanna-Rose 2006) (Figure 1).

We aimed to decipher the signaling mechanisms required downstream of LET-23 activation that act to counteract excitotoxic death and promote cell survival. We first investigated the role of signaling components known to mediate LET-23 signaling. Signaling via LET-60 Ras is required for multiple LET-23 functions (Beitel *et al.* 1990; Han and Sternberg 1991) and is specifically required for uv1 cell differentiation (Chang *et al.* 1999). Thus, we first asked if activation of LET-60 Ras could mimic the effect of *let-23* activation in preventing uv1 cell death. However, activation of LET-60 via the *n1046* gain-of-function allele did not prevent uv1 cell death in the *pnc-1* mutant or in NAM-supplemented cultures (Figure 1, A and B). Instead, we noted that some *let-60*(*n1046*gf) animals had more than two cell corpses per side of the uterus (Figure 1F). We hypothesized that these were extra uv1 cells specified from the uterine π lineage as the result of increased Ras signaling. Consistent with this hypothesis, *let-60*(*n1046*gf) single mutants have an increase in number of uv1 cells compared to wild-type animals (Table 2). We also examined *let-60*(*ga89*gf), a temperature-sensitive, gain-of-function allele (Eisenmann and Kim 1997). While only 28% (*n* = 25) of *let-23*(*sa62*gf) animals have one or more dying uv1 cells, 100% of NAM-treated *let-60*(*ga89*gf) animals have one or more dying uv1 cells at 15° (*n* = 66) and 25° (*n* = 49). Moreover, 20% of *let-60*(*ga89*gf) animals also have more than four dying uv1 cells at 25° (*n* = 49). These results demonstrate that overactivation of Ras sufficient to cause ectopic uv1 cell induction using two distinct gain-of-function alleles is insufficient to promote cell survival.

To further investigate any potential role of the Ras/MAPK pathway in mediating LET-23 cell survival functioning, we also examined animals in which the pathway is activated via heat shock–inducible coexpression of gain-of-function D-MEK and MPK-1, which encode MAP kinases that act downstream of Ras (Lackner *et al.* 1994; Lackner and Kim 1998). Activation of the pathway in this manner marginally promoted cell survival in NAM-treated animals (Figure 1B). We conclude that Ras/MAPK signaling may be involved but is likely not sufficient to substitute for LET-23 activity in cell survival signaling.

We next asked if activation of the signaling pathway involving the second messenger, IP_3_, which mediates the effect of *let-23*(*sa62*gf) in healthspan extension and ovulation (Clandinin *et al.* 1998; Yin *et al.* 2004; Iwasa *et al.* 2010), would mimic *let-23* cell survival effects. However, activation of the IP_3_ receptor using the gain-of-function allele *itr-1*(*sy290*gf) failed to mimic *let-23*(*sa62*gf) suppression of cell death (Figure 1A). Finally, we combined *let-60*(*n1046*gf) and *itr-1*(*sy290*gf) to test if there might be a requirement for simultaneous activation of these two pathways for *let-23*(*sa62*gf)-mediated cell survival signaling. The combination of these alleles had no effect on uv1 cell death (Figure 1A), but they did cooperate to produce ectopic uv1 cell induction (Table 2). We conclude that while activation of LET-23 is sufficient to prevent death of uv1 cells in the *pnc-1* mutant, activation of ITR-1 alone or in combination with LET-60 is not.

**Figure 2:**
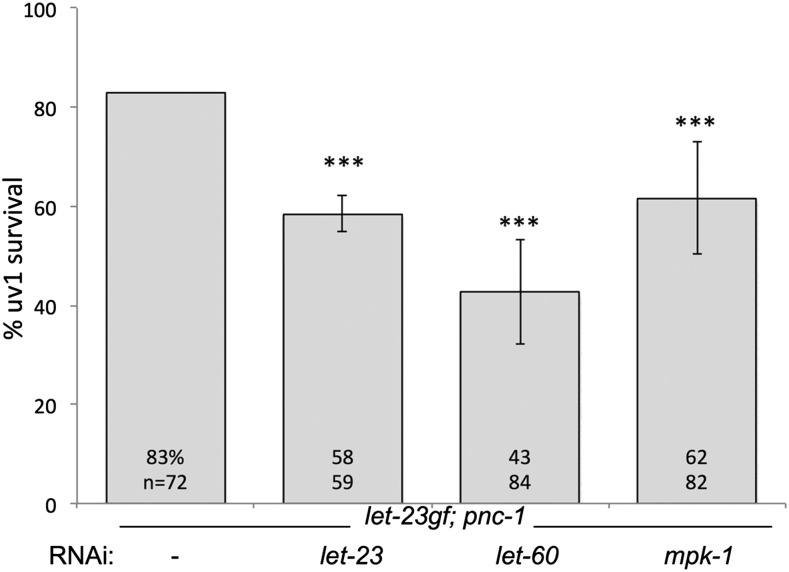
The LET-60 Ras MPK-1 MAPK signaling pathway is required for full cell survival signaling by LET-23. After RNAi of the indicated gene in *let-23*(*sa62*gf); *pnc-1* (strain HV330), animals that were no longer multivulva (demonstrating effectiveness of the RNAi) were scored for surviving uv1 cells. Percentage of uv1 cells surviving is plotted. The actual percentages and number of animals scored are indicated on each bar. Error bars are 1 SD. *** *P* < 0.001, calculated using Fisher’s exact test with Bonferroni correction considering three pairwise comparisons.

To determine if either *let-60* or *itr-1* has any function in *let-23*(*sa62*gf)-mediated suppression of cell death, we knocked down expression of these genes in the *let-23*(*sa62*gf); *pnc-1* animals and scored uv1 cell survival. *let-60* RNAi resulted in a decrease in cell survival as did *mpk-1* RNAi (Figure 2). These RNAi treatments gave as robust of an effect as *let-23* RNAi itself (Figure 2), which was surprisingly resistant to knockdown by RNAi, suggesting that LET-60 Ras and the downstream MAP kinase MPK-1 are at least partially required for LET-23 function in preventing cell death. The RNAi experiments and gain-of-function experiments together demonstrate that signaling via LET-60 Ras downstream of LET-23 is necessary but not fully sufficient for LET-23 signaling function in promoting uv1 survival. In contrast, we detected no role for *itr-1* or *plc-3*. uv1 cell survival remained robust in the *let-23*(*sa62*gf); *pnc-1* strain upon *itr-1* RNAi (86% survival, *n* = 42) or *plc-3* RNAi (87% survival, *n* = 46).

### RNAi screen for genes that mediate LET-23 cell survival signaling

**Figure 3:**
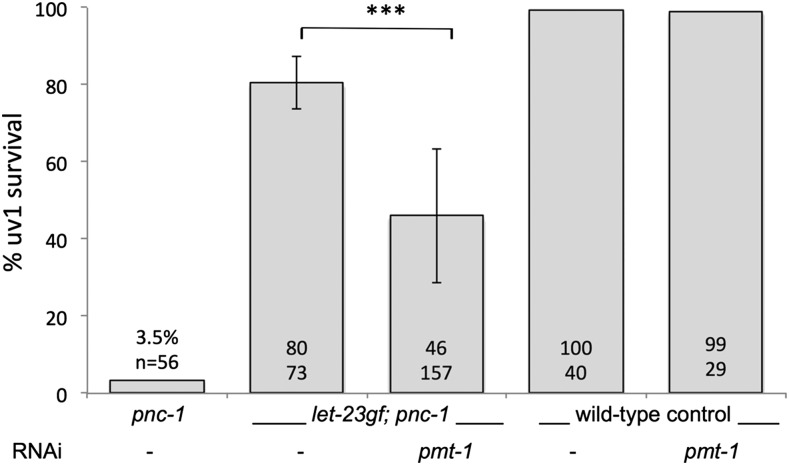
*pmt-1* is required for efficient survival of uv1 cells in the *let-23*(*sa62*gf); *pnc-1* background but does not affect differentiation or survival of uv1 cells in a wild-type background. Percentage of uv1 cells surviving is plotted for *pnc-1*(*pk9605*) (strain HV560) as well as *let-23*(*sa62*gf); *pnc-1* (strain HV776), and wild-type control (strain BL5715), and strains treated with RNAi targeting *pmt-1*. *pmt-1* RNAi experiments were performed in triplicate in the *let-23*(*sa62*gf) background. The average percentages and number of animals scored are indicated on or above each bar. Error bars are 1 SD. *** *P* < 0.001, calculated using Fisher’s exact test.

Because *let-60* appears to be required for *let-23*-mediated cell survival signaling but *let-60*(*n1046*gf) is not sufficient to mimic the effect of *let-23*(*sa62*gf), we hypothesized that other signaling pathways and mechanisms are acting downstream of, or in parallel to *let-60* to mediate *let-23* activity in uv1 cell survival. To identify these genes we carried out a targeted RNAi screen in *let-23*(*sa62*gf); *pnc-1* animals that carry a transgene causing GFP expression in uv1 cells. Knockdown of any gene that is required for *let-23* function in promoting uv1 cell survival is expected to result in increased cell death in *let-23*(*sa62*gf); *pnc-1* animals, and thus a decrease in number of healthy GFP-positive uv1 cells. To rule out RNAi clones that simply prevent or reduce uv1 specification and thus decrease the number of GFP-positive uv1 cells present, we eliminated genes whose RNAi resulted in reduced numbers of GFP-positive cells in a wild-type background. We assembled a list of candidate genes with known signaling roles that consisted primarily of kinases. A screen of 234 candidates (Table S1) revealed two positive genes, including *pmt-1*, which was serendipitously included among the kinase candidates, likely because of cross-contamination from an adjacent well in the library plate. Knockdown of *pmt-1* reduced *let-23*(*sa62*gf)-mediated uv1 survival by almost half in the *let-23*(*sa62*gf); *pnc-1* animals but did not affect uv1 specification in a wild-type background (Figure 3). We conclude that *pmt-1* is required for LET-23 to promote cell survival in *pnc-1* animals. Because of the robust effect of *pmt-1* RNAi and the novel and unexpected role for the gene, we chose to continue characterizing *pmt-1*.

### PtdCholine synthesis is necessary for LET-23-mediated suppression of cell death

**Figure 4:**
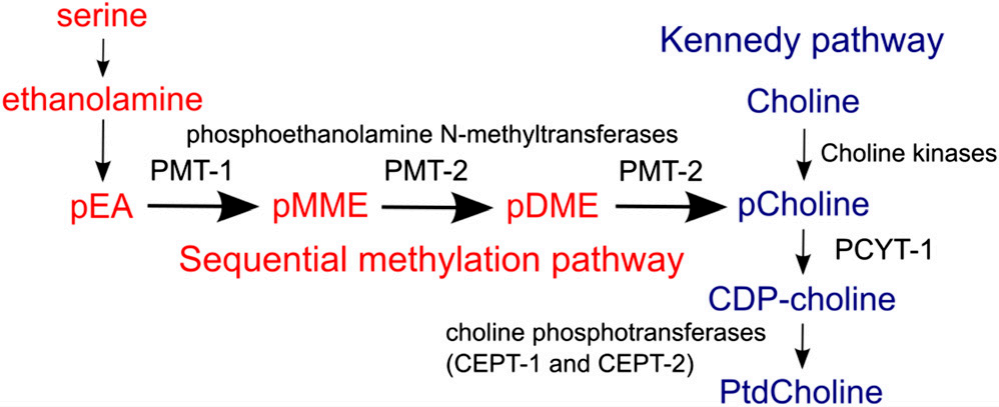
Sequential methylation (red) and Kennedy (blue) pathways for biosynthesis of phosphatidylcholine (PtdCholine) in *C. elegans*. The sequential methylation pathway starts with phosphoethanolamine (pEA) to produce phosphocholine (pCholine) through two methylation steps catalyzed by the phosphoethanolamine *N*-methyltransferases, PMT-1 and PMT-2 (Brendza *et al.* 2007). Phosphomonomethylethanolamine (pMME) and phosphodimethylethanolamine (pDME) are the methylated intermediates. pCholine is also produced by phosphorylation of choline by choline kinases. pCholine is converted to cytidine diphosphate (CDP)-choline by CTP:phosphocholine cytidylyltransferase PCYT-1. Finally, PtdCholine is produced by the choline-ethanolamine phosphotransferases CEPT-1 or CEPT-2 using diacylglycerol and CDP-choline as substrates.

**Figure 5:**
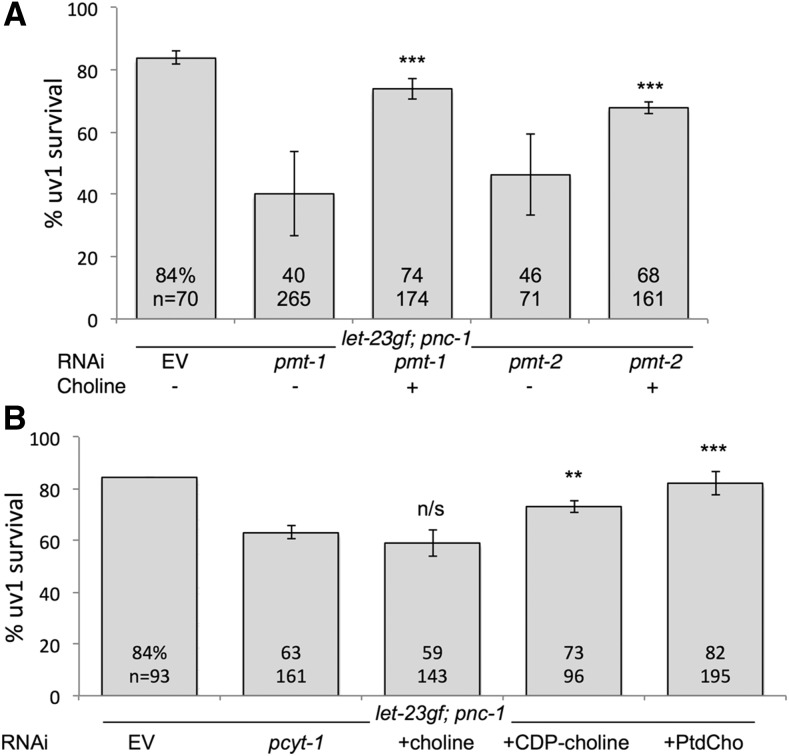
The sequential methylation and Kennedy pathways are required for efficient LET-23 survival signaling. (A) Both sequential methylation pathway genes *pmt-1* and *pmt-2* are required for LET-23-mediated survival signaling, but this requirement is bypassed by supplementation with exogenous 30 mM choline. (B) The Kennedy pathway gene *pcyt-1* is required for LET-23-mediated survival signaling in the *pnc-1* mutant (strain HV776). This requirement is bypassed by supplementation with exogenous 30 mM CDP-choline or 30 μM phosphatidylcholine but not 30 mM choline. Supplementation experiments were performed in triplicate. The average percentages and number of animals scored are indicated on each bar. Error bars are 1 SD. *** *P* < 0.001, ** *P* < 0.002, n/s, not significant, compared to nonsupplemented *pcyt-1* control using Fisher’s exact test.

PMT-1 is a phosphoethanolamine *N*-methyltransferase that adds a methyl group to phosphoethanolamine at the start of the sequential methylation pathway of phosphocholine (pCholine) synthesis (Brendza *et al.* 2007) (Figure 4). Given that PMT-1 functions within a defined biochemical pathway (Palavalli *et al.* 2006; Brendza *et al.* 2007), we wanted to determine if a specific metabolite from the sequential methylation pathway or the Kennedy pathway, which subsequently converts pCholine to PtdCholine (Figure 4), was required for *let-23* activity in preventing cell death. We first asked if PMT-2, a second phosphoethanolamine *N*-methyltransferase that acts after PMT-1 in the sequential methylation pathway, was also necessary for *let-23*(*sa62*gf)-mediated uv1 survival. Knockdown of *pmt-2* in the *let-23*(*sa62*gf); *pnc-1* background results in an increase in cell death similar to that of *pmt-1* RNAi (Figure 5A). We conclude that *pmt-2* is also required for *let-23* to promote uv1 survival. To test if *pmt-1* and *pmt-2* are required for function of *let-23* in other signaling events, we examined the effect of RNAi of these genes on vulval development and uv1 specification. *let-23*(*sa62*gf) causes both increased vulval induction (Katz *et al.* 1996) and ectopic uv1 specification (Table 2), but neither phenotype is affected by *pmt-1* or *pmt-2* RNAi (Table 3).

**Table 3:** Reduction of phosphocholine synthesis via *pmt* RNAi does not reduce LET-23 activity in vulval development or uv1 cell specification

	No. of Vulval Invaginations L4 Stage						No. of GFP-Positive uv1 Cells				
	1	2	3	4	*n*	Average	4	5	6	*n*	Average
Wild type*^a^*	50	0	0	0	50	1.00	30	0	0	30	4.00
*let-23*(*sa62*gf)*^b^*	2	21	10	1	34	2.29	49	8	1	58	4.17
*let-23*(*sa62*gf); *pmt-1* RNAi	0	13	37	8	58	2.91*	32	8	0	40	4.20
*let-23*(*sa62*gf); *pmt-2* RNAi	0	8	10	0	18	2.56	36	11	2	49	4.31

* *P* < 0.05, calculated using Student’s *t*-test.

a Strain BL5715.

b Strain HV760.

*pmt-1* and *pmt-2* RNAi animals have viability and fertility defects that can be rescued by supplementation with exogenous choline, an alternative substrate that can be used to synthesize pCholine via the Kennedy pathway (Figure 4) (Palavalli *et al.* 2006; Brendza *et al.* 2007). Therefore we next tested if exogenous choline was able to restore *let-23*-mediated uv1 survival to *let-23*(*sa62*gf); *pnc-1* animals treated with *pmt-1* or *pmt-2* RNAi. Addition of 30 mM choline to *pmt-1* or *pmt-2* RNAi plates significantly restored uv1 cell survival (Figure 5A), suggesting that pCholine, the metabolic product of the sequential methylation pathway, is required downstream of or in parallel to LET-23 in suppressing cell death.

A primary role of pCholine is the production of PtdCholine. To determine if PtdCholine production is required for LET-23 function in suppressing cell death, we next examined the functional role of the genes involved in the Kennedy pathway for production of PtdCholine from pCholine (Figure 4). PCYT-1 encodes the *C. elegans* CTP:phosphocholine cytidylyltransferase (Kennedy and Weiss 1956), and CEPT-1 and CEPT-2 encode choline-ethanolamine phosphotransferases (Xu *et al.* 1994). *pcyt-1* RNAi significantly reduced *let-23*(*sa62*gf)-mediated uv1 cell survival (Figure 5B) and this effect was reversed by supplementation with exogenous CDP-choline, the product of PCYT-1, but not by supplementation with choline (Figure 5B). Individual knockdown of *cept-1* (83% survival, *n* = 162) or *cept-2* (93% survival, *n* = 143), or knockdown of both genes together (82% survival, *n* = 85) did not affect uv1 survival. However, supplementation of PtdCholine reversed the effect of *pcyt-1* RNAi (Figure 5B), suggesting that production of the final product of the Kennedy pathway is the metabolite with relevant function in preventing uv1 cell death in response to LET-23 activity in the *pnc-1* mutants.

**Figure 6:**
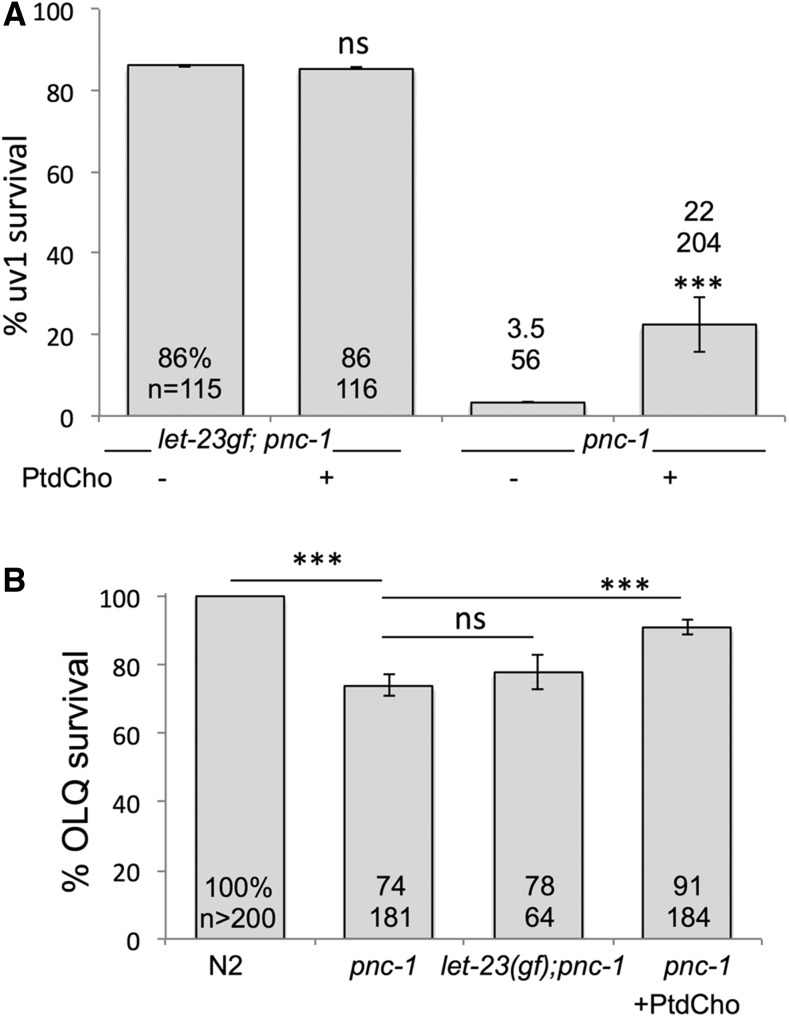
Exogenous phosphatidylcholine is sufficient to promote significant cell survival in an excitotoxicity model. (A) Supplementation with 30 µM phosphatidylcholine partially prevents uv1 death in *pnc-1*(*pk9605*) animals (strain HV560), but does not further increase survival in *let-23*(*sa62*gf); *pnc-1* animals (strain HV776). Error bars are 1 SD. (B) Supplementation with 30 µM phosphatidylcholine partially prevents OLQ death in *pnc-1*(*pk9605*) animals (strain HV560). The actual percentages and number of animals scored are indicated on or above each bar. Error bars are 95% C.I. ns, not significant; *** *P* < 0.001, calculated using Fisher’s exact test with Bonferroni correction considering five pairwise comparisons.

To test this model, we asked if boosting levels of PtdCholine would be sufficient to mimic *let-23*(*sa62*gf) activity. Supplementation of PtdCholine to *pnc-1*(*pk9605*) cultures resulted in a small but significant increase in uv1 survival (Figure 6A), whereas supplementation with CDP-choline did not (7% survival, *n* = 117). Supplementation of PtdCholine to *pnc-1*(*pk9605*) *let-60*(*n1046*gf) (strain HV662) cultures did not increase survival compared with *pnc-1*(*pk9605*) (18% survival, *n* = 232). A second cell type, called OLQ cells, is sensitive to NAM-induced excitotoxic death (Upadhyay *et al.* 2016). However, *let-23* is not reported to be expressed in OLQ cells (Van Buskirk and Sternberg 2007) and has no effect on OLQ cell survival (Figure 6B). As a further test of our model that PtdCholine production is protective against excitotoxic death in this model, we asked if PtdCholine supplementation could prevent OLQ death in the *pnc-1* mutant. Supplementation of PtdCholine to *pnc-1*(*pk9605*) cultures resulted in a small but significant increase in OLQ survival as well (Figure 6B). Our results are consistent with a role for the Kennedy pathway, and specifically PtdCholine, in mediating cell survival signaling.

## Discussion

Activation of LET-23 EGFR has a protective effect on uv1 cells that would otherwise die in response to OSM-9/OCR-4 TRPV channel-induced excitotoxicity. We show that the known signaling pathways downstream of LET-23 EGFR in *C. elegans* are differentially required to provide the protective effect. While *let-60* Ras is required for the full protective effect, we detected no role for signaling via ITR-1 IP_3_ receptor or PLC-3 phospholipase. Also, we found that our methods to increase activity of Ras or MAPK were not sufficient to promote full survival. The failure of constitutive Ras pathway signaling indicates that other unidentified signaling components are being used downstream of LET-23 in promoting cell survival. Alternatively, it remains possible that stronger LET-60 activity might prove sufficient for uv1 cell survival.

By screening for components required for LET-23-mediated survival signaling, we found that efficient PtdCholine biosynthesis is required for full survival signaling by LET-23. Moreover, supplementation of cultures with PtdCholine can protect *pnc-1* mutant cells from death, suggesting that EGFR regulation of lipid biosynthesis may underlie its survival function.

In the process of examining uv1 cell death, we also revealed that *let-60* activation is sufficient to induce ectopic specification of uv1 cells, a result that has not been previously noted. While *let-60* is known to be required for specification of uv1 cells, the ability of constitutive activation of either *let-60* or *let-23* to induce ectopic uv1 cells is novel. Interestingly, *itr-1*(*sy290*gf) does not mimic *let-60*(*n1046*gf) in this function but can enhance *let-60* activity in ectopic uv1 cell induction. The ability of LET-23 activation to induce ectopic uv1 cells is somewhat surprising, given that *let-23* expression has not been previously detected within cells of the uterine π lineage other than the presumptive uv1s (Van Buskirk and Sternberg 2007).

Regulation of lipids by EGFR has been noted previously in various systems. During *Drosophila* oogenesis, EGFR regulates expression of the gene *Cct1*, which is functionally required for ovarian morphogenesis and encodes the phosphocholine cytidylyltransferase orthologous to *C. elegans* PYCT-1 (Gupta and Schüpbach 2003). In other systems, EGFR also affects lipid biosynthesis via regulation of FASN activity (Bollu *et al.* 2014; Bian *et al.* 2015; Dahlhoff *et al.* 2015). Mutation of *Drosophila cct1* mutants reduces PtdCholine levels and negatively affects EGF and Notch signaling via regulation of endosome recycling (Weber *et al.* 2003). We directly examined whether the negative effect on LET-23 activity observed after inhibition of PtdCholine biosynthesis genes might be because of such an upstream effect on the LET-23 EGFR receptor. If interfering with PtdCholine biosynthesis similarly inhibits LET-23 activity in our experiments, we might expect all functions of LET-23 to be compromised upon *pmt-1* RNAi. In contrast, we found that while *pmt-1* RNAi interfered with LET-23-induced uv1 cell survival, it had no effect on LET-23-induced vulval induction (Table 3). Thus, our results are more consistent with a model whereby PtdCholine biosynthesis is required downstream of or in parallel to LET-23 specifically in cell survival signaling.

While both *pmt* methyltransferase genes in the sequential methylation pathway for pCholine biosynthesis as well as *pcyt-1*, the subsequent gene in the Kennedy pathway, are all similarly required for full LET-23-mediated survival signaling, we detected no effect of either *cept-1* or *cept-2* RNAi. Yet, supplementation with PtdCholine restored survival signaling activity to the *let-23*(*sa62*gf); *pnc-1*(*pk9605*); *pcyt-1* RNAi animals and even provided significant protective effect on its own to the *pnc-1* mutants, suggesting that the final product of the Kennedy pathway, PtdCholine, is a relevant metabolite required for promoting cell survival although the mechanism by which exogenous PtdCholine restores uv1 cell survival is not known. The reason for the lack of detected role for *cept-1* or *cept-2* is unclear. However, the experiments could be complicated by full or partial redundancy of the *cept* genes. The double RNAi experiment may not be sufficient to remove enough enzyme activity to curtail function. While we favor an interpretation that posits a protective role for PtdCholine, we have not ruled out CDP-choline as a relevant product. CDP-choline did not, however, provide protection as a sole supplement to *pnc-1* mutants.

How might a cell-signaling pathway prevent uv1 excitotoxic cell death? We propose that the effect of LET-23 is likely cell autonomous given that uv1 cells, which are known to express LET-23 (Chang *et al.* 1999), are protected, but OLQ cells, which are also sensitive to NAM-induced excitotoxic death (Upadhyay *et al.* 2016), are not reported to express *let-23* and are not protected by *let-23*(*sa62*gf) (Figure 6B). Given the known roles for PtdCholine derivatives in cell signaling, and the evidence that *Drosophila* *Cct1* may act cell nonautonomously (Gupta and Schüpbach 2003), it remains possible that a lipid-derived signal is important for cell death protection. However, RNAi knockdown of *pld-1*, the gene encoding phospholipase D that converts PtdCholine to phosphatidic acid (Matthies *et al.* 2006), has no effect on rescue (90.1% survival, *n* = 88).

While the answer to the question about the molecular mechanism of death prevention is unclear, we can speculate that protection from death could occur by interfering directly with TRPV channel gating, assembly, or activity, or by interfering with downstream events that promote execution of the cell death program. Given that NAM-induced toxicity can be rapidly induced in the uv1 cells within a minute of exogenous application of NAM (Upadhyay *et al.* 2016), and that PNC-1 acts cell nonautonomously (Crook *et al.* 2014), we speculate that a direct effect on the channel, which in turn prevents the death trigger, would be the most effective way to prevent death. Also, because we have identified a cell membrane constituent as a primary molecular component required for LET-23 cell survival activity, we speculate that this is consistent with direct effects on the ion channel. Moreover, lipids are known modifiers of TRPV channel activity (Zygmunt *et al.* 1999; Kahn-Kirby *et al.* 2004). Further work will be needed to clarify if LET-23 is affecting PtdCholine synthesis directly and if PtdCholine levels are important cell autonomously in the uv1 cells. However, our data are consistent with a speculative model that LET-23-mediated effects on PtdCholine biosynthesis affect aspects of channel activity in the membrane. It will be of interest to measure effects of LET-23-mediating signaling on PtdCholine levels and to examine effects of further manipulation of PtdCholine levels.

## Supplementary Material

Supplemental Material
