# Supplementary material for: Epidermal Growth Factor Receptor Cell Survival Signaling Requires Phosphatidylcholine Biosynthesis
Source: G3 (Bethesda). 2016 Sep 7;6(11):3533–40. doi: 10.1534/g3.116.034850 (PMC5100852; doi:10.1534/g3.116.034850)
Supplement: Supplemental Material [file supp_6_11_3533__index.html]

EGFR Cell Survival Signaling Requires Phosphatidylcholine Biosynthesis — Epidermal Growth Factor Receptor Cell Survival Signaling Requires Phosphatidylcholine Biosynthesis — Supplemental Material 

# Epidermal Growth Factor Receptor Cell Survival Signaling Requires Phosphatidylcholine Biosynthesis

## Supplemental Material for Crook, *et al*, 2016

**Files in this Data Supplement:**

- Table S1 - Genes screened via RNAi. (.pdf, 452 KB)
