# Supplementary material for: Epidermal Growth Factor Receptor Cell Survival Signaling Requires Phosphatidylcholine Biosynthesis
Source: G3 (Bethesda). 2016 Sep 7;6(11):3533–40. doi: 10.1534/g3.116.034850 (PMC5100852; doi:10.1534/g3.116.034850)
Supplement: Supplemental Material [file supp_g3.116.034850_TableS1.pdf]

S1 Table. Genes screened via RNAi

Gene	uv1 survival	n	Gene	uv1 survival	n
**Positive candidates [a]**					
pmt-1 **(ZK622.3)**	.48	87	*cdk-2* (Y39G8C.2)	.69	120
**False positives [b]**					
bub-1	.28	73	*plk-1*	.02	53
cdk-1	.02	79	*tlk-1*	.13	38
ndk-12	.64	49	*wee-1.3*	.16	59
ngp-1	.67	128			
**Negative candidates [c]**					
AH6.1	0.83	23	F54G8.2	0.88	26
B0025.1	0.86	26	F54H5.2	0.96	27
B0207.7	0.87	17	F55A12.3	0.88	15
B0218.5	0.88	26	F55G1.8	0.96	20
B0252.1	0.89	22	F56A4.g	0.89	25
B0285.10	0.84	24	F59A3.8	0.90	17
B0285.8	0.96	24	F59A6.1	0.85	25
B0285.9	0.94	27	F59B1.8	0.83	26
B0334.8	0.91	14	F59E12.3	0.88	30
B0414.7	0.92	25	H01G02.2	0.88	19
B0495.2	0.84	29	H06H21.8	0.85	17
B0496.3	0.96	24	H12I13.d	0.89	27
B0511.4	0.96	20	H25P06.2a	0.93	23
B0523.1	0.94	25	H37N21.1	0.93	28
C01G6.8	0.88	22	K03E5.3	0.88	28
C01H6.9	0.90	17	K06A1.6	0.90	24
C03C10.1	0.84	19	K06H7.8	0.91	25
C03C10.2	0.93	25	K07F5.4	0.92	22
C04G2.10	0.85	18	K08B12.5	0.86	24
C04G2.2	0.87	19	K08B4.5	0.93	14
C04G6.1	0.90	25	K08F8.1	0.75	32
C04H5.4	0.91	19	K09B11.1	0.94	20
C05C12.1	0.88	21	K09B11.5	0.93	20
C06A12.4	0.86	37	K09C6.7	0.92	24
C06E8.3	0.89	18	K09C6.8	0.88	13
C07G1.3	0.87	25	K11H12.9	0.87	27
C08F8.6	0.87	19	M03A1.1	0.94	25
C08H9.5	0.82	19	M03C11.1	0.94	13
C08H9.8	0.84	29	M04C9.5	0.85	28

C09B9.4	0.84	17	M176.6	0.83	46
C09D4.3	0.94	13	M176.9	0.84	23
C09G4.2	0.84	28	M7.7	0.89	20
C10C6.1	0.83	15	PAR2.3	0.89	20
C14A4.13	0.85	15	R02C2.2	0.75	13
C16A11.3	0.85	25	R03D7.5	0.98	13
C17C3.11	0.88	22	R05H5.4	0.92	15
C17F4.6	0.95	25	R09D1.12	0.87	25
C18H7.4	0.95	24	R107.4	0.79	44
C24A1.3	0.83	21	R11E3.1	0.80	28
C25A8.5	0.89	19	R134.1	0.83	25
C25H3.1	0.91	26	R134.2	0.84	22
C26E6.1	0.85	26	R13F6.7	0.84	24
C27D8.1	0.90	18	R13H9.5	0.89	22
C28A5.6	0.88	22	R13H9.6	0.81	17
C28D4.2	0.86	23	R151.4	0.91	24
C29F9.7	0.78	24	R166.5	0.88	19
C29H12.5	0.83	28	T04B2.2	0.91	14
C32D5.2	0.93	25	T05E8.2	0.89	25
C33F10.2	0.97	22	T06C10.3	0.86	11
C34B2.3	0.85	31	T06C10.6	0.88	23
C34C6.5	0.81	22	T07A9.3	0.89	21
C34F11.5	0.91	26	T09B4.7	0.82	14
C34G6.5	0.90	27	T10B11.2	0.85	31
C35D10.4	0.85	18	T11F8.4	0.84	14
C36B1.10	0.86	31	T13H10.1	0.86	21
C39H7.1	0.86	21	T15B12.2	0.81	24
C41C4.4	0.88	18	T17A3.8	0.89	19
C44C8.6	0.84	51	T17E9.1	0.84	22
C45G9.1	0.90	21	T19D12.5	0.91	23
C46C2.1	0.87	17	T20F10.1	0.88	30
C48B6.6	0.85	24	T21G5.1	0.84	24
C49C3.2	0.89	21	T22B11.3	0.93	19
C49C8.1	0.90	13	T25B9.4	0.85	12
C49H3.1	0.89	19	T25B9.5	0.95	10
C55B7.10	0.92	22	T26C12.4	0.91	29
C55C3.4	0.91	19	T27C10.5	0.91	32
C56C10.6	0.91	22	T27E9.3	0.90	24
D1044.1	0.82	25	W01B6.2	0.92	24
D1044.3	0.82	19	W01B6.5	0.95	22
D2024.1	0.85	17	W02B12.12	0.84	23
D2045.5	0.83	21	W02B3.2	0.93	26
D2045.7	0.92	15	W03A5.1	0.80	20

E02D9.1	0.90	24	W03F11.2	0.93	20
EEED8.9	0.91	26	W03F8.2	0.97	17
F01D4.3	0.94	20	W03G1.6	0.80	60
F08B4.3	0.93	20	W03G9.5	0.82	27
F09C12.2	0.94	25	W04B5.5	0.91	27
F11E6.8	0.90	21	W06F12.3	0.88	19
F12A10.2	0.89	30	W08D2.8	0.81	20
F12F3.2	0.83	31	Y106G6A.1	0.91	31
F18F11.4	0.77	14	Y106G6D.4	0.83	57
F22B3.8	0.83	26	Y106G6E.6	0.82	31
F22D6.1	0.92	23	Y111B2A.b	0.89	14
F22D6.5	0.87	23	Y119D3_455.b	0.83	18
F22E5.3	0.80	25	Y11D7A.8	0.92	22
F23C8.7	0.93	22	Y18H1A_68.g	0.81	32
F23C8.8	0.94	31	Y38H8A.3	0.88	24
F25F2.1	0.78	15	Y38H8A.4	0.85	21
F26A1.3	0.88	26	Y39G8B.f	0.88	26
F26A1.4	0.89	24	Y43C5B.2	0.94	20
F26E4.5	0.85	31	Y47G6A_245.h	0.84	26
F28B12.3	0.91	24	Y47G6A_246.a	0.83	25
F31E3.2	0.85	24	Y48G1B_55.a	0.81	26
F32B6.4	0.95	21	Y48G1C_55.a	0.86	27
F33E2.2	0.85	31	Y4C6A.k	0.93	18
F35H8.7	0.93	30	Y52D3.1	0.91	16
F36H12.8	0.93	21	Y54E10B_152.b	0.83	12
F36H12.9	0.89	21	Y65B4A_185.a	0.80	21
F37E3.3	0.92	13	ZC123.4	0.93	27
F39H11.3	0.86	25	ZC239.7	0.90	25
F41G3.5	0.85	27	ZC416.4	0.89	26
F42A10.4	0.84	24	ZC581.1	0.88	18
F42G8.3	0.94	17	ZC581.2	0.89	27
F42G8.4	0.83	15	ZC581.7	0.92	25
F44D12.11	0.90	21	ZC581.9	0.87	23
F45H7.4	0.86	28	ZK1067.1	0.83	50
F46F5.2	0.92	27	ZK177.2	0.86	24
F48G7.12	0.94	16	ZK354.2	0.82	26
F48G7.3	0.88	18	ZK370.5	0.94	21
F49B2.5	0.88	26	ZK593.9	0.90	13
F49C12.7	0.88	14	ZK622.1	0.91	27
F49C5.4	0.83	18	ZK632.3	0.87	23
F49E11.1	0.94	18	ZK896.8	0.93	18
F52B5.2	0.88	29	ZK930.1	0.89	11
F52C12.2	0.85	21	ZK938.5	0.89	22

F52F12.3	0.84	32	ZK970.5	0.87	15
F53C3.1	0.91	25	ZK970.6	0.86	24
F53G12.6	0.93	21			

[a] decreased uv1 cell survival in *let-23(gf) pnc-1(lf)* but not in a wild-type background

[b] decreased uv1 cell survival in *let-23(gf) pnc-1(lf)* and in a wild-type background

[c] no effect on uv1 cell survival in *let-23(gf) pnc-1(lf)*
